# Complex synapses as efficient memory systems

**DOI:** 10.1186/1471-2202-16-S1-F1

**Published:** 2015-12-18

**Authors:** Marcus K Benna, Stefano Fusi

**Affiliations:** 1 Center for Theoretical Neuroscience, Columbia University, College of Physicians and Surgeons, New York, NY 10032, USA

## 

The molecular machinery underlying memory consolidation at the level of synaptic connections is believed to employ a complex network of highly diverse biochemical processes that operate on a wide range of different timescales. An appropriate theoretical framework could help us identify their computational roles and understand how these intricate networks of interactions support synaptic memory formation and maintenance.

Here we construct a broad class of synaptic models that can efficiently harness biological complexity to store and preserve a huge number of memories, vastly outperforming other synaptic models of memory. The number of storable memories grows almost linearly with the number of synapses, which constitutes a substantial improvement over the square root scaling of previous models [1,2], especially when large neural systems are considered. This improvement is obtained without significantly reducing the initial memory strength, which still scales approximately like the square root of the number of synapses.

This is achieved by combining together multiple dynamical processes that operate on different timescales, to ensure the memory strength decays as slowly as the inverse square root of the age of the corresponding synaptic modification. Memories are initially stored in fast variables and then progressively transferred to slower ones. Importantly, in our case the interactions between fast and slow variables are bidirectional, in contrast to the unidirectional cascades of previous models.

The proposed models are robust to perturbations of parameters and can capture several properties of biological memories, which include delayed expression of synaptic potentiation and depression, synaptic metaplasticity, and spacing effects. We discuss predictions for the autocorrelation function of the synaptic efficacy that can be tested in plasticity experiments involving long sequences of synaptic modifications.
